# Generation of a tumor- and tissue-specific episomal non-viral vector system

**DOI:** 10.1186/1472-6750-13-49

**Published:** 2013-06-04

**Authors:** Rudolf Haase, Terese Magnusson, Baowei Su, Florian Kopp, Ernst Wagner, Hans Lipps, Armin Baiker, Manfred Ogris

**Affiliations:** 1 Department of Pharmacy, Center for Drug Research, Pharmaceutical Biotechnology, Ludwig-Maximilians-University, Munich, Germany; 2 Centre for biomedical Education and Research, Institute for Cell Biology, University of Witten/Herdecke, Witten, Germany; 3 Bavarian Health and Food Safety Authority, Oberschleissheim, Germany

**Keywords:** Tumor Targeting, pDNA, Episomal, AFP, SM22, Tissue-specific Replication

## Abstract

**Background:**

A key issue for safe and reproducible gene therapy approaches is the autologous and tissue-specific expression of transgenes. Tissue-specific expression in vivo is either achieved by transfer vectors that deliver the gene of interest into a distinct cell type or by use of tissue-specific expression cassettes. Here we present the generation of non-viral, episomally replicating vectors that are able to replicate in a tissue specific manner thus allowing tissue specific transgene expression in combination with episomal replication. The episomal replication of the prototype vector pEPI-1 and its derivatives depends exclusively on a transcription unit starting from a constitutively active promoter extending into the scaffold/matrix attachment region (S/MAR).

**Results:**

Here, we exchanged the constitutive promoter in the pEPI derivative pEPito by the tumor specific alpha fetoprotein (AFP) or the muscle specific smooth muscle 22 (SM22) promoter leading to specific transgene expression in AFP positive human hepatocellular carcinoma (HUH7) and in a SM22 positive cell line, respectively. The incorporation of the hCMV enhancer element into the expression cassette further boosted the expression levels with both promoters. Tissue specific-replication could be exemplary proven for the smooth muscle protein 22 (SM22) promoter in vitro. With the AFP promoter-driven pEPito vector hepatocellular carcinoma-specific expression could be achieved in vivo after systemic vector application together with polyethylenimine as transfection enhancer.

**Conclusions:**

In this study we present an episomal plasmid system designed for tissue specific transgene expression and replication. The human AFP-promoter in combination with the hCMV enhancer element was demonstrated to be a valuable tissue-specific promoter for targeting hepatocellular carcinomas with non-viral gene delivery system, and tissue specific replication could be shown in vitro with the muscle specific SM22 promoter. In combination with appropriate delivery systems, the tissue specific pEPito vector system will allow higher tissue-specificity with less undesired side effects and is suitable for long term transgene expression in vivo within gene therapeutical approaches.

## Background

Sustained and tissue specific transgene expression are key issues for the development of successful gene therapy approaches. In principle, tissue specificity can be achieved either by gene delivery systems which explicitly deliver the gene of interest into a distinct cell type or tissue [1] or by the use of tissue-specific expression cassettes [2]. For sustained transgene expression, integrating vectors, like viruses [3] or nonviral systems based on integrases or transposons have been utilized [4,5]. To avoid the risk of insertional mutagenesis, as it has been observed for example with retrovirus [6], nonintegrating vectors like episomally retained systems can be of use. The first prototype episomal vector pEPI-1 was cons-tructed by Piechaczek and colleagues [7] carrying a chromosomal scaffold/matrix attachment region (S/MAR) derived from the β-interferon gene [8] within a commercially available plasmid backbone; pEPI-1 is able to replicate episomally with a copy number of approximately 5–10 molecules per cell and is stably retained without applying selection pressure allowing long-term expression of transgenes or shRNA’s [7,9-11]. Since integration of this vector into the host cell chromosome was never observed, side effects caused by gene disruption or activation would not be expected, but cannot be fully excluded. In contrast to episomal plasmid replicons based on animal viruses, pEPI-based episomal DNA vectors do not contain any transactivating viral proteins such as the SV40 large T-antigen or the EBV EBNA-1 protein, which may contribute to cellular immortalization [12]. An upstream transcription unit regulated by the CMV immediate early promoter (CMV-IEP) and directed into the S/MAR is necessary for episomal replication of pEPI [10,13,14]. Our approach here was to modify this vector system by exchanging the constitutive promoter with tissue- or tumor-specific ones to generate a vector, which will episomally express the transgene in the target cell type only, but being lost in all other cell types. Recently, similar effects with pEPI vectors utilizing chemically inducible promoters could be shown [15].

Only a small percentage (between 0.5 and 5%) of cells transiently transfected with pEPI-1 develops stable clones, also when pEPI-1 plasmids are isolated from already established cell clones and reintroduced into cells or if pEPI-1 episomes are administered as HPV16 based pseudoviral particles. This demonstrates that the primary DNA sequence is not sufficient for stable establishment, but other epigenetic factors are involved in this process [14,16]. A further improvement of pEPI-1 concerning transgene expression and episome establishment was previously published as pEPito [17]. pEPito consists of an identical transcription unit as pEPI, but contains a CpG-low vector backbone achieving higher expression levels and episomal establishment rates compared to pEPI. Initially, the constitutive CMV or EF1α promoter was used in pEPito vectors. In this study, we replaced them with tissue specific promoters: i) the alpha fetoprotein (AFP) promoter drives expression of AFP in the embryonic liver and in hepatocellular carcinomas [18], ii) the SM22 promoter has been described as tissue-specific promoter for smooth muscle cells [19,20]. A tissue-specific AFP-dependent expressing pEPito derivate achieved liver carcinoma specific expression in vitro and in vivo. For the SM22-promoter driven pEPito derivative we present the tissue-specific replication of a S/MAR based vector in vitro. To cope with the relatively low activity of tissue specific promoters, we utilized the hCMV upstream of the AFP or SM22 promoter. As the episomal maintainance of the pEPI-based vectors are dependent on the transcription into the S/MAR region [10], the replication of those vectors in cells without any activity for the promoter should not occur.

## Results

### The AFP promoter is more active in HUH7 hepatocellular carcinoma compared to HPGL promoter and APOE enhancer

**Figure 1:**
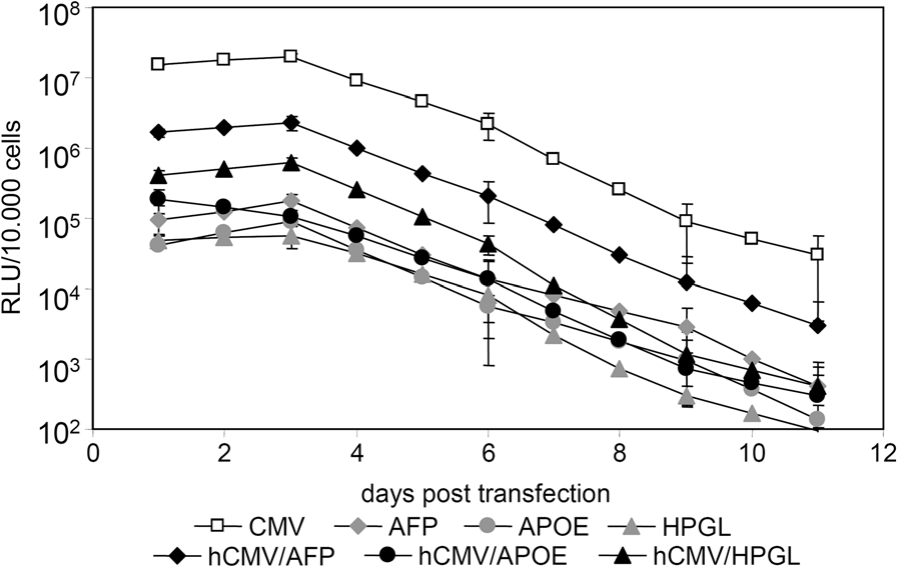
**Influence of the hCMV enhancer on liver promoter mediated transfection in hepatocellular carcinoma cells.** 50,000 HUH7 cells seeded in 24-well plates were transfected with HD-O polyplexes containing the indicated plasmid (1.5 μg plasmid/well) and the luciferase activity per 10,000 cells determined at indicated time points; n = 2. Open symbols: CMV promoter; gray symbols: liver promoter; full symbols: liver promoter plus hCMV enhancer.

Three liver specific promoters, alpha-fetoprotein promoter (AFP), haptoglobin promoter (HPGL) and the apolipoprotein E enhancer (APO E) were evaluated for their use as liver specific episomal vectors. The AFP and HPGL promoters were obtained by PCR amplification of genomic DNA obtained from HEK293 cells. Promoter selection and primer choice were done under use of TiProD. To enhance promoter activity, a human cytomegalovirus (hCMV) enhancer was cloned in front of the promoter sequence. As depicted in Figure 1, all constructs featuring an hCMV enhancer element achieve higher expression levels compared to the enhancer free construct. The CMV driven vector pEPito-CMV-EGFPLuc served as positive control achieving the highest luminescence signal, followed by the hCMV/AFP-construct, the hCMV/HPGL-construct and the much weaker respective APOE-derivative. As expected, the APOE enhancer without the hAAT promoter turned out to be inefficient for driving transgene expression. Monitoring transgene expression over a period of 11 days exhibits decreasing expression signals over time, as no selection pressure was put on the transfected cells. As the hCMV enhancer/AFP-promoter combination achieved highest activity of all liver specific promoter elements tested, it was selected for the further experiments.

### AFP promoter is most active in AFP-producing cell lines, but also achieves weak expression levels in non-liver derived cell lines

**Figure 2:**
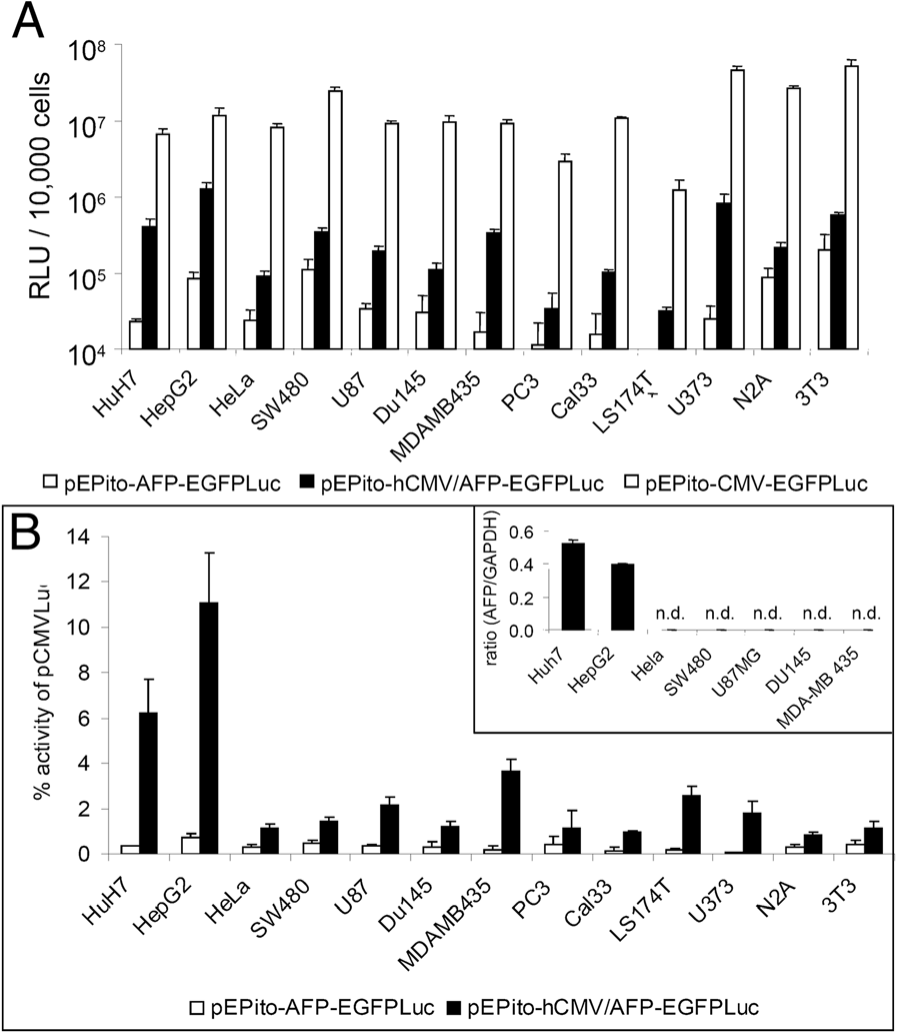
**Expression activity of CMV and AFP promoter driven plasmids in human and murine cell lines.** Indicated cell lines were seeded in 96-well plates (10,000 cells/well seeded), transfected with LPEI polyplexes (200 ng Plasmid/well) for four hours and the luciferase activity per 10,000 cells measured 24 h thereafter (n = 5 + stddev) (**A**) Absolute luciferase activity (RLU). Background activity of untransfected cells was below 1000 RLU for all cell lines evaluated. (**B**) Relative luciferase activity of plasmids pEPito-AFP-EGFPLuc and pEPito-hCMV/AFP-EGFPLuc normalized to the expression activity of pEPito-CMV-EGFPLuc in the respective cell line; insert in (**B**): mRNA level of AFP mRNA normalized to the housekeeper mRNA GAPDH in indicated cell lines (n = 2 + stddev., n.d.: not detectable).

Plasmids pEPito-CMV-EGFPLuc, pEPito-AFP-EGFPLuc and pEPito-hCMV/AFP-EGFPLuc were transfected into 13 different human and murine cancer cell lines (hepatoma, cervix carcinoma, colon carcinoma, glioma, prostate carcinoma, melanoma, squamous cell carcinoma, neuroblastoma) and a murine fibroblast cell line (Figure 2). In all cell lines transfected, the AFP plasmid without enhancer element resulted in only very low to undetectable levels of luciferase activity, whereas the highest levels of luciferase activity were achieved by the CMV driven plasmid (Figure 2A). The latter value (10^7^ RLU/10,000 cells seeded) corresponds to a transfection efficiency based on percentage of transgene positive cells ranging from 20-60% using EGFP encoding, CMV driven plasmids (M. Ogris et al., unpublished observation). The hCMV enhancer increased the expression activity of the AFP driven plasmid, although there appeared to be strong variability between the cell lines. As there can be a large variability in terms of transfectability of various cell lines with transfection enhancers like polyethylenimine, we normalized the luciferase activity to the values achieved with the CMV driven plasmid (Figure 2B). Without hCMV enhancer, the relative activity varied between 0.05 and 0.7%. When using the hCMV enhancer, in the hepatoma cell lines HUH7 and HepG2 the activity was boosted 18-fold and 15-fold, achieving 6% (HUH7) resp. 12% (HepG2) activity relative to CMV respectively. In all other cell lines, only 1-2% of CMV activity was obtain, with the exception of MDA-MB-435 human melanoma (3.6%). The weak background expression in all cell lines might be linked to the general tumor characteristics of immortalized cell lines. To investigate the role of AFP expression for AFP promoter driven transgene expression, we performed quantitative real time analysis on endogenous AFP mRNA levels using GAPDH as internal standard (Figure 2B, insert). Only in the reportedly AFP positive cell lines HUH7 and HepG2 considerable levels of AFP mRNA were detected, whereas in all other cell lines tested they were undetectable (below background values, i.e. not detectable after 45 rounds of PCR amplification). Hence we conclude that the endogenous AFP levels and the activity of the used AFP promoter show a positive correlation, although some minor background activity is observed in AFP negative cell lines. All data obtained so far indicate a functional usage of the hCMV/AFP hybrid promoter element with satisfactory specificity and sensitivity for hepatocellular carcinomas.

### AFP promoter is active in a xenograft model of HUH7 in mice

**Figure 3:**
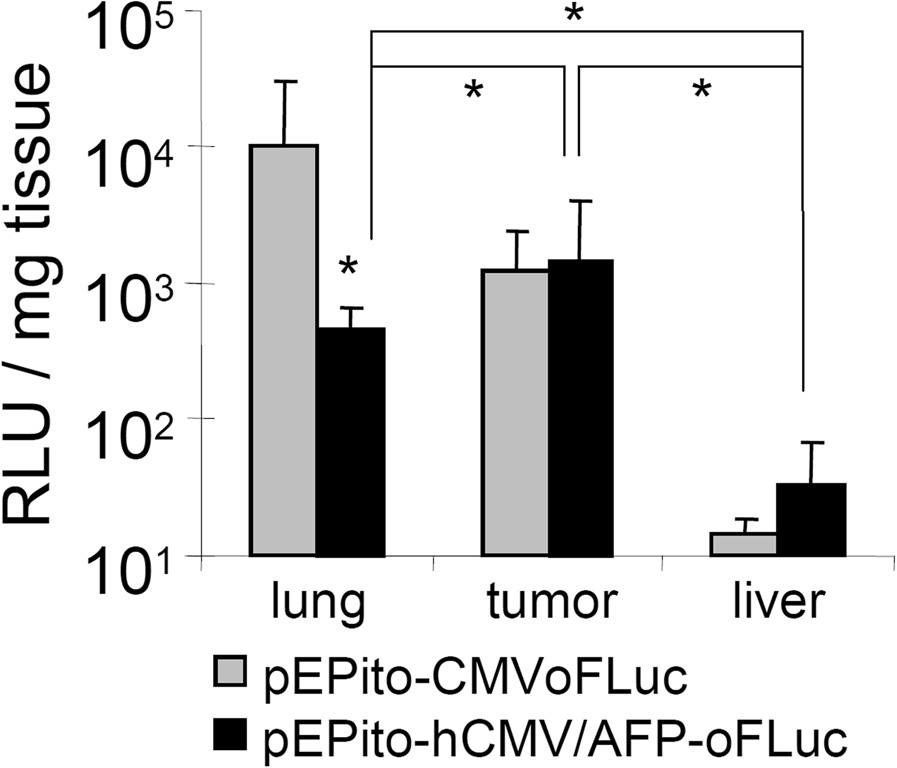
**Hepatoma specific transgene expression with pEPito-hCMV/AFP-oFLuc *in vivo. ***NMRI nu/nu mice bearing subcutaneous HUH7 human hepatoma tumors were injected intravenously with LPEI polyplexes (N/P 6) formed either with pEPito-hCMV/AFP-oFLuc or pEPito-CMV-oFLuc at a dose of 2.5 mg/kg (n = 6 per group). Luciferase activity (RLU/mg tissue) is shown in lysates of lung, tumor and liver 24 h after transfection; grey bars: pEPito-CMV-oFLuc, full bars: pEPito-hCMV/AFP-oFLuc; n = 6; * p < 0.05 (*U*-test, Mann–Whitney; lung signal pEPito-hCMV/AFP-oFLuc vs. pEPito-CMV-oFLuc).

To investigate the in vivo expression pattern of our newly developed hCMV/AFP construct, we utilized the HUH7 xenograft tumor model in immune compromised nude mice. The EGFPLuc transgene was replaced by an expression optimized Firefly Luciferase (oFLuc), which is reported to achieve better in vivo signals in comparison to the standard luciferase [21]. We also observed significantly higher luciferase activity with oFLuc-plasmids in vitro when compared to the normal EGFP luciferase fusion protein (data not shown). NMRI nu/nu mice carried subcutaneously implanted HUH7 tumors in the flank, which are usually well vascularized and in principle accessible for polycation condensed plasmids after intravenous application [22]. As a transfection reagent, we used LPEI. LPEI polyplexes are known to rapidly aggregate in the blood stream leading to high transgene expression in the lung [23]. Here we aimed at investigating the de-targeting effects on lung expression when using the AFP promoter. Polyplexes were prepared with LPEI at an N/P ratio of 6 in a glucose buffer (HBG) and injected via the tail vein at a dose of 2.5 mg/kg based on pDNA (Figure 3). Two days after transfection, animals were subject to analysis of luciferase activity by bioluminescense imaging (BLI) and after three days luciferase activity was quantified in tumor- and tissue lysates. As already observed for other luciferase encoding plasmid with constitutive promoters, pEPito-CMV-oFLuc based polyplexes led to predominant activity in the lung area, whereas activity in all other areas was rather low and not always detectable by BLI. In sharp contrast, lung activity was strongly reduced with pEPito-hCMV/AFP-ofLuc polyplexes, and in some animals a weak signal was observed in the tumor area. To obtain quantitative data on luciferase activity, animals were sacrificed at day three after transfection and luciferase activity measured in lysates of organs and tumor. With the CMV driven plasmid, a considerable luciferase activity was observed in lung tissue, whereas the tumor expression was approx. eightfold lower. In sharp contrast to the AFP driven plasmid, luciferase activity in lung was 20-fold lower, whereas the luciferase expression in tumor was unaffected. Hence we conclude the general functionality of the hCMV/AFP-promoter for HCC-specific expression, both in vitro and in vivo*.*

### Specific replication of a SM22 driven pEPito vector in a muscle derived cell line

**Figure 4:**
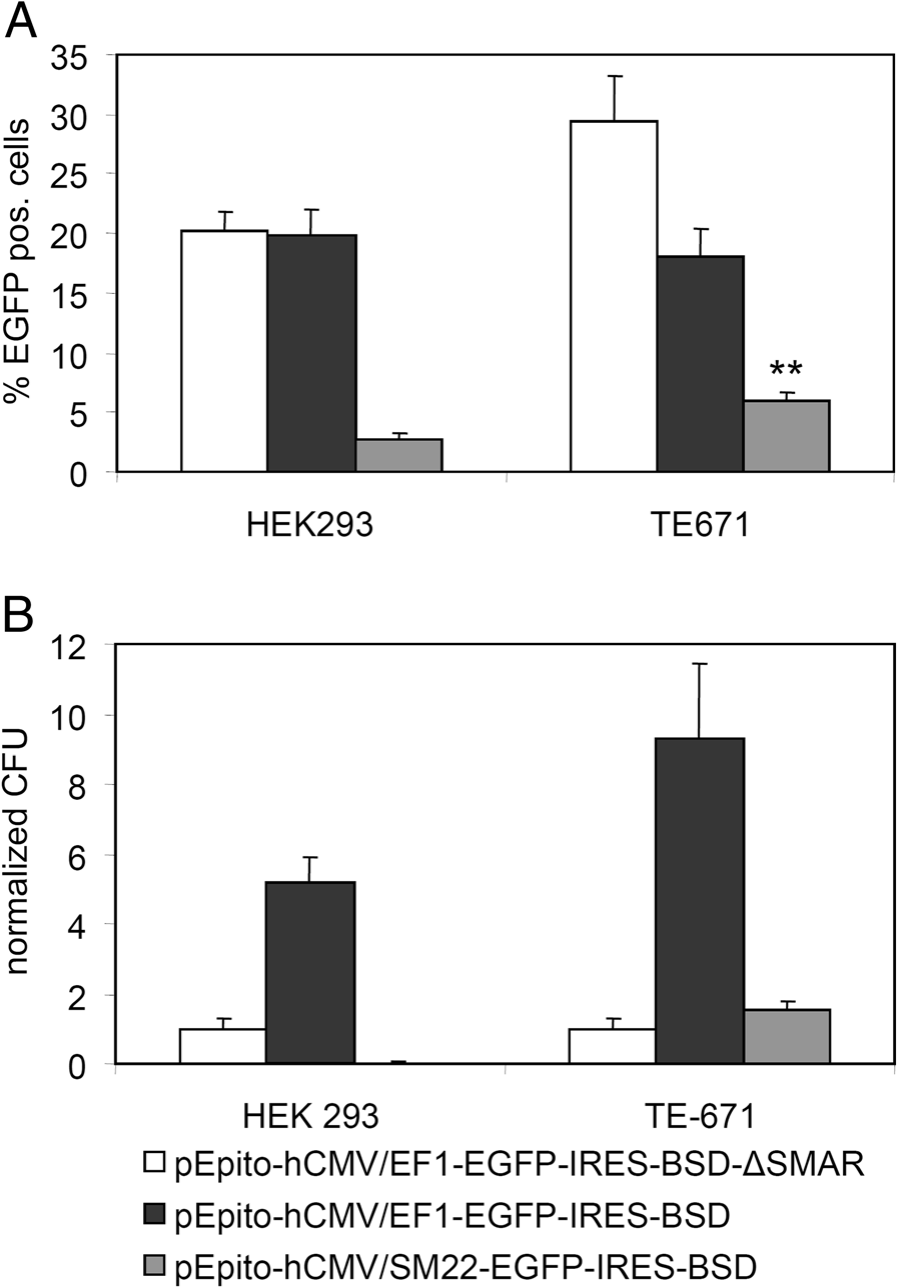
**Transient transfection efficiency and establishment efficiency of episomes in HEK293 and TE-671.** Cells were transfected with the indicated plasmid and Fugene 6 as transfection reagent (**A**) Percentage of EGFP positive cells 48 hours after transfection; n = 6 + stddev, ** p = 0.003 (*U*-test, Mann–Whitney, pEPito-hCMV/SM22-IRES-BSD transfection HEK293 vs. TE-671) (**B**) Relative colony-forming efficiency of stably selected clones normalized to the establishment efficiency obtained with (pEPito-1- [hCMV/EF1αP]- [EGFP-IRES-BSD]-ΔSMAR). n = 6 + stddev.

For tissue specific expression and replication in healthy, adult tissue, we evaluated the human transgelin promoter (synonym SM22), which is reported to be active in muscle cells [19,20,24]. As establishment of the episomes is easily detectable under selection, we applied vectors with a blasticidin expression cassette for our experiments. HEK293 were used as negative control, whereas the muscle derived cell line TE-671 served as the positive control. 150,000 cells were transfected with 0.25 μg DNA (using Fugene6 as transfection reagent) and two days post transfection cells were analyzed for the percentage of EGFP positive cells (Figure 4A) and EGFP expression levels per EGFP positive cell (mean fluorescence intensity, MFI, data not shown). All experiments were performed by transfecting equal mass amounts of vector DNA instead of equal molar ratios of vector molecules, as we already did in our previous work [17]. This should exclude potential side effects of stuffer DNA, like sonicated salmon sperm DNA or any other small plasmid DNA with varying CpG contents. In line with our previous observations [17], pEPito-hCMV/EF1-EGFP-IRES-BSD and pEPito-hCMV/EF1-EGFP-IRES-BSD-ΔSMAR resulted in approx. 20% EGFP positive cells, whereas with pEPito-hCMV/SM22-EGFP-IRES-BSD >3% were EGFP positive. The expression level (MFI) in HEK 293 for the hCMV/SM22 promoter construct was also much weaker (MFI < 2,500, less than 8% in direct comparison) than the levels for the hCMV/EF1 driven pEPito (>20,000). In contrast, the hCMV/SM22 construct was significantly more active in TE-671 cells, >6% of cells were EGFP positive, corresponding to one third of the activity of pEPito-hCMV/EF1-EGFP-IRES-BSD (18%). The expression level (MFI) was at about 15% of the constitutive active promoter. The higher percentage of EGFP-positive cells observed after transfection with pEPito-hCMV/EF1-EGFP-IRES-BSD-ΔSMAR can be explained by the smaller size of this plasmid. A similar behaviour of the ΔSMAR construct was previously observed in NIH3T3 cells [17]. After 14 days of selection with blasticidin S a further FACS analysis was performed to analyze the percentage of EGFP positive cells and their MFI value (data not shown). Here, plasmids lacking the SMAR-element were not able to achieve stable expression, whereas with S/MAR bearing constructs prolonged expression was achieved. Furthermore the SM22-promoter achieved much better expression rates and expression levels in the TE-671 cell line than in the HEK 293 cells when compared to the EF1α-promoter. We omitted a ΔSMAR control vector for the tissue-specific derivative as a logical consequence to our previous experiments. If the pEPito-hCMV/EF1-EGFP-IRES-BSD-ΔSMAR is not able to achieve persistence, the weaker expressing tissue-specific ΔSMAR-construct would not perform better.

With the help of the colony forming assay the establishment rate of stable episomes and background integration values was evaluated (Figure 4B). Colony formation units (CFU) were calculated by normalizing the colony number to the value obtained with pEPito-hCMV/EF1-EGFP-IRES-BSD-ΔSMAR as recently described [17]. In HEK 293 cells, the hCMV/SM22-construct resulted only very few background colonies, whereas in TE-671 cells the same construct achieved more colonies than the pEPito-hCMV/EF1-EGFP-IRES-BSD-ΔSMAR. In comparison, the hCMV/SM22 driven construct succeeded in almost 20 fold more colonies in the TE-671 cell line than in the HEK293 cells when compared to the hCMV/EF1-driven pEPito. Therefore we conclude the functionality of the tissue-specific expression of the SM22 promoter in the muscle derived TE-671 cells. The minimal expression levels of the SM22 promoter in the HEK293 cells (Figure 4A) did not achieve episomal retention of this plasmid. It is known, that transcription in the SMAR is a prerequisite for episomal establishment [15]. We confirmed the establishment as an episome by rescue experiments (Table 1). Only the pEPito-hCMV/EF1-EGFP-IRES-BSD could be rescued in both cell lines. The negative control, pEPito-hCMV/EF1-EGFP-IRES-BSD-ΔSMAR did not give any positive result, which is in line with previous experiments [7,17]. pEPito-hCMV/SM22-EGFP-IRES-BSD could only be rescued from genomic DNA of the stably selected TE-671 cells, indicating that this construct was maintained here as an epsiome. On average, about hundred positive clones were obtained from cells carrying pEPito-hCMV/EF1-EGFP-IRES-BSD, whereas from TE-671 carrying pEPito-hCMV/SM22-EGFP-IRES-BSD approximately 25 positive colonies were obtained. Rescued plasmid were identified by different restriction digests. pEPito-hCMV/EF1-EGFP-IRES-BSD-ΔSMAR did not result in any bacterial retransformants. Taken together, the SM22-promoter achieved better expression rates and expression levels in the TE-671 cell line, confirming its usefulness as a tissue-specific promoter for muscle-specific cells. For the first time we could also show its function as a tissue-specific promoter in a pEPI-deriviative in terms of episomal establishment.

**Table 1:** Rescue experiments with genomic DNA from stably transfected cells

**Plasmid**	**HEK293**	**TE-671**
pEPito-hCMV/EF1-EGFP-IRES-BSD	3/3	3/3
pEPito-hCMV/EF1-EGFP-IRES-BSD-ΔSMAR	0/9	0/9
pEPito-hCMV/SM22-EGFP-IRES-BSD	0/9	3/3

First number: number of rescue experiments where plasmid DNA could be rescued without any rearrangement from the isolated chromosomal DNA. Second number: total number of performed rescue experiments. ‵0′ indicates that no clones could be obtained in any rescue experiment.

## Discussion

In this manuscript we present the first description of an episomal and tissue-specific replicating SMAR-based vector. Combining the properties of an episomally replicating, non-integrating vector system with tissue specific promoters should in principle give a high level of biosafety when compared for example to integrating viral vector systems. Nevertheless, the low efficiency of non-viral systems can be a limiting factor. Recently, we improved the existing episomal vector system pEPI by replacing the backbone with a CpG depleted backbone and the promoter sequence with the strong, non-silenced constitutive promoter for elongation factor 1α [17]. The novel vector series termed pEPito was superior in expression strength both in vitro and in vivo and exhibited strongly improved establishment efficiency as a non-integrated episome leading to long term gene expression. The aim of our first experiments was to develop a pEPito vector with a strong and specific promoter for liver and liver derived cancer cells, which will allow the expression of high transgene levels when used within this episomal vector construct. From our previously published work we knew that the SMAR element often leads to reduced transient expression levels when compared to the ΔSMAR control (see e.g. [17]). As tissue specific promoters are usually weaker then strong, constitutive promoters like CMV or EF1α, a careful selection process is necessary to identify promoters with adequate specificity, but also sufficient strength for pEPito constructs. In all experiments, transfections with either constitutive or tissue specific promoters were run in parallel and not using them as internal controls with a different transgene. Although useful in other experimental setups, using a plasmid with strong constitutive promoter can potentially interfere with the transfection efficiency obtained (by competing for transcription factors or at mRNA levels for translation factors), but also in rare recombination events, as several plasmid parts (poly A signal, bacterial promoter, resistance cassette) are similar to the tested plasmid. Alpha-fetoprotein is a major fetal serum protein in mammals and has it functions in ligand transport and maintaining physiologic osmolarity [25]. The AFP gene is transcribed at high levels in the yolk sac and fetal liver, where the transcription declines dramatically after birth, resulting in barely detectable AFP levels in the liver by 4 weeks of age [26]. In hepatocellular cancer, AFP becomes active again, a mechanism which is not yet fully clarified yet. Silencer sequences play a critical role in AFP promoter activity, but also regulatory regions or growth related genes [27] or hepatocyte nuclear factor 3 [28] were reported to be involved in this process. The most used variant of the AFP-promoter element is of murine origin including its enhancer, which exhibits (including enhancer elements) a size >2600 bp [18,29]. Our 600 bp AFP promoter was amplified from human genomic DNA, and together with the hCMV enhancer element was only approximately 850 bp in size. The APO E enhancer is usually used in combination with the hAAT promoter for liver specific expression for factor IX in gene therapy [30,31]. Here we omitted the hAAT promoter element to achieve higher specificity, as the original hybrid APO E-hAAT promoter achieved too high activity in HEK 293 cells in pre-experiments (data not shown). The haptoglobin promoter was chosen as it appeared to be very liver specific according to TiProD [32]. Its gene product, haptoglobin, is an abundant haemoglobin-binding protein present in the plasma [33]. On the human hepatocellular cell line HUH7, all three liver specific promoters resulted in a weak transgene expression level far below 1% of the CMV driven activity. This is in line with observations made with other tissue specific promoters, like the murine AFP promoter [18] or muscle specific promoter elements [34]. We and other groups could show that the hCMV enhancer element boosts transgene expression of ubiquitous promoter both in vitro and in vivo, for example in lung [35] or liver [36]. For this purpose, we cloned the hCMV enhancer element upstream of the respective promoters. Although the activity of all three promoters was increased by the hCMV enhancer element, only the hCMV/AFP combination gave satisfyingly high levels also several days after transfection (Figure 1), so it was used in the successive experiments. To ensure that with the hCMV enhancer element specificity is retained, control transfections were carried out in AFP negative cell lines both of human and murine origin (Figure 2). After normalizing for the activity obtained with the CMV driven plasmid it could be observed that the AFP positive cell lines HUH7 and HepG2 were most susceptible to AFP mediated transgene expression, whereas in almost all other cell lines the hCMV enhancer element just boosted basal expression levels in general (Figure 2B). QPCR studies confirmed that the HCC cell lines HUH7 and HepG2 were positive for AFP expression levels [18,29,37,38]. Although no detectable levels of AFP mRNA in the human melanoma cell line MDA-MB-453, this cell line was at least to a certain extent susceptible to hCMV/AFP mediated transfection. Approx. 3.6% of the CMV driven transgene expression were achieved. This can be due to certain ‘leakiness’ of the AFP promoter or the hCMV enhancer element, as it has been observed for viral vectors with tissue or tumor specific promoters [39]. Apparently the leakiness of our hybrid promoter element also depends on the cell type of tumor type. We can furthermore not exclude that the observed basal activity of the AFP promoter might be a indication of the tumor characteristics of all immortalized cell lines.

To prove the applicability of the AFP driven pEPito vector in vivo, nu/nu mice bearing subcutaneously implanted HUH7 tumors were systemically treated with polyplexes based on LPEI. Such LPEI polyplexes usually rapidly aggregate with blood components like erythrocytes and a considerable portion accumulates in the first vascular bed encountered, namely the lung, were they also lead to efficient transgene expression [23]. Nevertheless, transgene expression can also be found in other organs, like liver [40], and subcutaneously implanted tumors [22,41]. CMV driven transgene expression usually peaks 12-24 h after delivery, e.g. after LPEI polyplex mediated delivery in lung tissue (M. Ogris et al., unpublished, and [42]) or after hydrodynamic plasmid delivery to the liver [36,43] and rapidly declines thereafter. Measuring luciferase activity by BLI 48 h after delivery, a high signal was observed in the lung area with the CMV driven plasmid, and after 72 h there was still a considerable signal remaining. Luciferase activity could not be detected with the AFP driven construct by BLI, and was 20-fold lower after 72 h in the lysates. As the tumor expression was unchanged, we apparently could improve the in vivo specificity >20 fold. Hence we conclude that with hCMV/AFP driven transgene expression a considerable tumor specificity accompanied by strong activity in the target tissue (HCC) can be achieved. Although useful for proof of principle studies like presented here, unmodified LPEI is not the ideal carrier for targeting transgene expression to the tumor after systemic injection: In our experiment presented here, the level of luciferase activity in tumors strongly varied between individual animals (CMV driven plasmid: 4 high, 2 low, AFP driven plasmid: 3 high, 3 low). This variation in transfectability does not correlate with size the of HUH7 tumors (ranging from 4–10 mm in diameter in this experiment), as subcutaneous HUH7 tumors are already hypervascularised at small diameter (3 mm and above, M. Ogris et al., unpublished observations), and blood vessels are enlarged and highly leaky [44]. We expect an influence of functional blood flow, which can strongly vary between tumors, but also between different time points were polyplexes are applied. Here we rather aimed at proving the de-targeting effect from lung. Other, better suited delivery systems based on LPEI developed in our group (including shielding domains to reduce aggregation and target domains to direct transgene expression to epidermal growth factor receptor overexpressing cells) are far superior for systemic HUH7 tumor targeting, but only show very low or absent activity in non-target organs including lung [45,46].

Showing the principle usability of the AFP promoter to efficiently drive transgene expression within a pEPito vector in vitro and in vivo, but also some leakiness due to activity on AFP negative cell lines, we examined the tissue-specific replication of a SMAR-based vector within a second promoter setup using similar backbone and enhancer. For this purpose the muscle specific SM22 promoter (human variant synonym transgelin), was chosen. This promoter was reported to be tissue-specific and to achieve robust transgene expression levels. With the SM22 promoter we also expected a higher degree of specificity and less leakiness then with AFP, as it has also been described by other groups [39]. In vivo replication for S/MAR-based vector could so far only be shown after sperm-mediated gene transfer [47], but not yet with transient transfection using episomal vectors [17,43], unless recently a selection and/or a growth advantage was included into the episomal plasmids by coexpressing Bcl-2 [48]. Nevertheless, it has to be noted that Bcl-2 overexpression itself is known to be antiapoptotic and this gene is upregulated in tumors. Due to these difficulties and as the SM22 promoter is also rather weak when compared to constitutive promoters and to AFP promoter (R. Haase, unpublished observations), we decided to study cell specific replication within an in vitro setting. As the constitutive control, the EF1α promoter was selected, which leads to long lasting transgene expression within pEPito vectors and is not prone to rapid silencing like the CMV immediate early promoter element [17,36]. As expected, transfection efficiency was high both in HEK293 and TE-671 cells, and in TE-671 cells the efficiency with the ΔSMAR construct was even higher. This is in line with our previously published data, and is due to the fact that the transcription of the S/MAR sequence decreases expression efficiency in certain cell lines [7,10,43,49]. Although at lower efficiency then achieved with the EF1α driven vector, SM22 driven expression was significantly higher in TE-671 cells when compared to HEK293 pointing at the specificity of this construct (Figure 4A). The establishment of stable clones with the EF1α driven pEPito was strongly depending of the presence of the S/MAR element in both cell lines. The SM22 only developed stable clones in TE-671 cells at lower efficiency when compared to the EF1α driven pEPito. As the transcription into the SMAR is crucial for the establishment of episomes [15], the strength of constitutive promoters influences the extent of the expression from the established clones [17]. These facts are also known for minicircles (J. Bode, personal communication, and ref. [50]). It seems to be logical, that much weaker promoters than the constitutive CMV, SV40 or EF1α promoters exhibit lower establishing rates, which would also explain the lower episome establishment of the muscle-specific pEPito derivative. Background integration for S/MAR-based vector can still not be fully excluded, but was reported to be repressed [51]. The small amount of stably transfected HEK293 cells with the pEPito-hCMV/SM22-construct (less than 2% of the number of the ΔSMAR construct (Figure 4B)) showed moderate expression levels (data not shown), but as the plasmid could not be rescued from this cell line, this indicates an integrated status of the plasmid. Apparently the background integration is dependent of the promoter expression strength, as the promoter drives the expression and selection unit of these vectors. We further conclude that for every successful rescue experiment, replicated plasmid DNA could be accounted, as otherwise all experiments would have shown positive colonies. Hence the number of established clones of the SM22-promoter driven construct in the TE671 cell line represent a low, but reproducible frequency of episomal establishment. To highlight the effect of the SMAR element, we have normalized the data to the EF1α driven plasmid lacking the SMAR element. The rescue experiments (Table 1) verified the episomal status of the tissue-specific pEPito in the respective cells, but also confirmed the correlation between promoter strength and episomal establishment rate [17]: with the constitutive and strong EF1α promoter, about hundred positive bacterial colonies were found per experiment, both with DNA extracts from HEK293 and TE671 cells, whereas with SM22 driven pEPitos this number was significantly lower (approx. 25 colonies).

## Conclusions

In this study we present an episomal plasmid systems designed for tissue specific transgene expression and replication. The human AFP-promoter in combination with the hCMV enhancer element was demonstrated to be a valuable tissue-specific promoter for targeting hepatocellular carcinomas with non-viral gene delivery system, and tissue specific replication could be shown in vitro with the muscle specific SM22 promoter. In combination with appropriate delivery systems, the tissue specific pEPito vector system will allow higher tissue-specificity with less undesired side effects and is suitable for long term transgene expression in vivo within gene therapeutical approaches.

## Methods

### Vector construction

An overview of all vectors used within this study is shown in Table 2. The pEPito-CMV-EGFPLuc, pEPito-hCMV/EF1-EGFP-IRES-BSD and pEPito-hCMV/EF1-EGFP-IRES-BSD-ΔSMAR were described before [17]. The human AFP-, HPGL- and SM22-promoter were amplified by PCR from genomic DNA of 293 HEK cells. All promoter sequences were obtained using TiProD [32]. In general, these sequences are the first 600 bp upstream of their natural gene product. The primers used contained PciI (5′) and NheI (3′) restriction sites. The APOE enhancer element (kindly provided by Mark Kay) was also amplified by PCR. Resulting PCR-fragments were subcloned into the pGEM-T-easy vector (Promega, Mannheim, Germany) and respective clones further characterized by sequencing (AGOWA, Berlin, Germany). Clones were digested with PciI and NheI and the respective fragments cloned into pEPito-CMV-EGFPLuc or pEPito-CMV-EGFP-IRES-BSD, which were digested with the same enzymes, thus creating pEPito-AFP-EGFPLuc, pEPito-APOE-EGFPLuc, pEPito-HPGL-EGFPLuc and pEPito-SM22-EGFP-IRES-BSD. The hCMV enhancer was amplified by PCR from pEPito-hCMV/EF1-EGFP-IRES-BSD and additional restrictions sites were added (NcoI and PciI). The pEPito-AFP-EGFPLuc, pEPito-APOE-EGFPLuc, pEPito-HPGL-EGFPLuc and pEPito-SM22-EGFP-IRES-BSD were digested by PciI and the amplified PCR fragment of the hCMV enhancer was ligated into the PciI site. The resulting constructs pEPito-hCMV/AFP-EGFPLuc, pEPito-hCMV/APOE-EGFPLuc, pEPito-hCMV/HPGL-EGFPLuc and pEPito-hCMV/SM22-EGFP-IRES-BSD were sequenced to verify the correct orientation of the hCMV enhancer. pMOD-ZGFP (Cayla Invivogen, France) was digested with AvrII and BamHI and the fragment was cloned into the vector backbone of pEPito-CMV-EGFPLuc with NheI and BglII restricted resulting in pEPito-CMV-ZGFP. The pEPito-CMV-ZGFP was digested with BglII and NheI and the oFluc fragment of pV2011-oFL (kindly provided by Dr. Brian Rabinovich, [21]) after digestion with BglII and AvrII was ligated into the vector backbone, thus creating pEPito-CMV-oFLuc. The same procedure was carried out for pEPito-hCMV/AFP-EGFPLuc to generate pEPito-hCMV/AFP-oFLuc. All constructs were propagated in E.coli DB3.1λpir [52].

**Table 2:** Promoter, transgene, size and selection option of all plasmids used in this study

**Plasmid**	**Promoter**	**Transgene**	**Size (bp)**	**Selection**
pEPito-CMV-EGFPLuc	CMV-IEP	EGFPLuc	7005	-
pEPito-AFP-EGFPLuc	AFP	EGFPLuc	6922	-
pEPito-hCMV/AFP-EGFPLuc	hCMV/AFP	EGFPLuc	7251	-
pEPito-APOE-EGFPLuc	APOE	EGFPLuc	6715	-
pEPito-hCMV/APOE-EGFPLuc	hCMV/APOE	EGFPLuc	7044	-
pEPito-HPGL-EGFPLuc	HPGL	EGFPLuc	6922	-
pEPito-hCMV/HPGL-EGFPLuc	hCMV/HPGL	EGFPLuc	7251	-
pEPito-AFP-oFLuc	AFP	oFLuc	6350	-
pEPito-hCMV/AFP-oFLuc	hCMV/AFP	oFLuc	6679	-
pEPito-CMV-oFLuc	CMV-IEP	oFLuc	6431	-
pEPito-hCMV/EF1-EGFP-IRES-BSD	hCMV/EF1	EGFP-IRES-BSD	5680	BSD
pEPito-hCMV/EF1-EGFP-IRES-BSD-ΔSMAR	hCMV/EF1	EGFP-IRES-BSD	3719	BSD
pEPito-hCMV/SM22-EGFP-IRES-BSD	hCMV/SM22	EGFP-IRES-BSD	6022	BSD

### Cell culture

Human HEK293 embryonic kidney cells (ATCC CRL-1573), TE-671 rhabdomyosarcoma (ACC 263), Cal33 tongue squamous cell carcinoma (ACC 447), SW480 colon carcinoma (ACC 313), HeLa cervix carcinoma (ATCC CCL-2) and Neuro 2A murine neuroblastoma (ACC 148) were cultured in DMEM (1 g glucose/l) completed with 10% FCS. U87MG human glioblastoma (ATCC HTB-14) were grown on collagen G (Biochrom) coated plates in DMEM/10% FCS. Human hepatocellular carcinoma cell lines HuH7 (JCRB0403) and HepG2 (ATCC HB-8065) and human melanoma cell line MDA MB-435 (ATCC HTB-129) were cultivated in DMEM/Ham’s F-12 medium 1:1 supplemented with 10% FCS and 2 mM stable glutamine. Du145 human prostate carcinoma line (ATCC HTB-81), LS174T human colon carcinoma (ATCC CL-188) and NIH3T3 murine fibroblasts were cultivated in RPMI 1640 (Biochrom) medium supplemented with 10% FCS. U373MG glioblastoma (ATCC HTB-17) were cultured in DMEM (5 g glucose/l) supplemented with 20% FCS. All cultured cells were grown at 37°C in 5% CO_2_ humidified atmosphere.

### EGFP transfection and FACS analysis

For transfection experiments with EGFP encoding plasmids, 1.5 × 10^5^ cells were seeded into a 6 well plate (BD Falcon, USA) 24 hours prior to the experiment and thereafter transfected using 0.25 μg vector DNA per well using Fugene6 (Roche, Germany) as transfection reagent according to the manufacturer’s instructions. Two days (48 hours) post transfection cells were trypsinized, resuspended in phosphate buffered saline (PBS) (Invitrogen, Germany), and divided into two aliquots. One aliquot was analyzed for EGFP expression using a FACS Canto II flow cytometer (Becton Dickinson, Germany). The other aliquot was cultured in the presence of blasticidin (7 μg/ml) (PAA, Austria). After 12 days of selection, stably selected (mixed-clone) cells were again analyzed for EGFP expression.

### Luciferase assay

Cells were seeded 24 h before transfection and the cell medium was exchanged for fresh growth medium (with serum) directly prior transfection. Polyplexes were formed in HEPES-buffered saline (HBS, 20 mM HEPES, 150 mM NaCl, pH 7.1) with pDNA and the transfection reagent HDO solution at a C/P (conjugate to plasmid, w/w) ratio of 2 at a final pDNA concentration of 20 μg/ml followed by 20 minutes incubation at room temperature. After 4 h transfection the solution was exchanged for fresh cell medium. For measuring luciferase activity, cells were trypsinised, 10,000 cells lysed in 0.5x lysis buffer (Promega, Mannheim, Germany) and analyzed by luciferase-assay [53]. Background activity of untransfected cells was <1,000 RLU.

### Xenograft nude mice model, in vivo transfection/transduction and in vivo bioluminescence

In vivo experiments were carried out in principle as described [22]. In brief, six week old female Rj:NMRI nu/nu mice (Janvier, Le Genest-Saint-Isle, France) received 5 x 10^6^ HUH7 cells resuspended in 100 μl PBS subcutaneously into the flank. After reaching a tumor size of 8 mm (2–3 weeks after inoculation) LPEI based polyplexes were formed at N/P 6 (molar ratio of nitrogen in LPEI to phosphate groups in plasmid DNA) with a final concentration of 200 μg/mL pDNA in HEPES-buffered glucose (HBG, 20 mM HEPES, 5% glucose w/v, pH 7.1) and injected intravenously at a DNA dose of 2.5 mg/kg. One, two and three days after injection animals were monitored by in vivo bioluminescence imaging (BLI) after intraperitoneal injection of 6 mg Na-luciferin (Promega, Mannheim, Germany) dissolved in 100 μl PBS. BLI was performed with an IVIS lumina system (CaliperLS, Mannheim, Germany). At day three after polyplex application, animals were euthanized, indicated organs and tumors explanted, lysed and the luciferase activity determined from an aliquot of the lysate.

Animals were housed in individually vented cages with a 12 h light/dark cycle and unlimited access to food and water. All animal experiments were approved by the local ethics committee (Regierung von Oberbayern) and carried out according to the German law for protection of animals (Tierschutzgesetz).

### Colony-forming assay

For colony forming assays, transfected cells were split from 6 well plates into 75 cm^2^ flasks at 48 hours post transfection. Splitting of cells was performed at serial dilutions (1:1, 1:10, and 1:100). After a total of 12 days of selection with blasticidin colonies were fixed with 4% paraformaldehyde (Sigma, Germany) in PBS, counterstained with methylene blue (Sigma, Germany) and counted.

### Isolation of genomic DNA from cell lines

For isolation of genomic DNA from transfected and stably selected cell lines, cells were trypsinized, resuspended in PBS, and counted. Genomic DNA was isolated from 10^7^ cells using the QiaAMP DNA Mini Kit (Qiagen, Germany) according to the manufacturer’s instructions.

### Quantitative real-time PCR

Total RNA was isolated using miRCURY RNA Isolation Kit (Exiqon, Vedbaek, Denmark) and transcribed with the Transcriptor High Fidelity cDNA Synthesis Kit (Roche, Mannheim, Germany) according to manufacturer’s protocols. Quantitative real-time PCR was performed using UPL Probes (Roche, Mannheim, Germany) and LightCycler 480 Probes Master (Roche, Mannheim, Germany) on a LightCycler 480 system (Roche, Mannheim, Germany). The following primer sequences were used for the human alpha-fetoprotein (NM_001134.1): AFP left: tgtactgcagagataagtttagctgac and AFP right: tccttgtaagtggcttcttgaac. AFP mRNA levels were normalized to GAPDH as control using Human GAPDH Reference Gene Assay (Roche, Mannheim, Germany). Experiments were done in triplicates and the obtained average C_T_ values were normalized to control as ΔC_T_. Expression changes in the target gene were analyzed as ratio AFP/GAPDH (2^-ΔCT^).

### Bacterial rescue experiments

To verify the episomal status of pEPito vectors within transfected and stably selected mammalian cell lines, bacterial rescue experiments were performed by chemical transformation of 10 μl isolated genomic DNA (approximately 500 ng) into chemical competent *E.coli* DB3.1λpir [52]. Transformed bacteria were selected on LB-plates containing ampicillin. Plasmid DNA was prepared from transformed bacteria using the Qiaprep Spin Miniprep Kit (Qiagen, Germany) according to the manufacturer’s instructions. The integrity of the rescued plasmids was checked by restriction analysis and gel electrophoresis. For rescue experiments of cell culture materials, chromosomal DNA of stably selected mixed clones was isolated three times independently and transformed into bacteria. Resulting bacterial clones were analyzed for the integrity of their isolated plasmids by three different restriction digests with XhoI (dual cutter), PciI and NheI (both single cutters) and BglII and BamHI (both single cutters). Only if the rescued and retransformed plasmids showed the same digestion pattern as the original plasmid, the rescue was counted as successful, In case no colonies could be obtained from the initial transformations, this procedure was repeated twice. For a complete negative rescue the DNA was isolated three times and each DNA isolation was transformed three times into the bacterial host strain.

## Abbreviations

AFP: Alpha-fetoprotein; APOE: Apolipoprotein E; bp: Basepair; BSD: Blasticidin S deaminase; CMV: Cytomegalovirus; CpG: Cytosinephosphatidyl-Guanosine; dpi: Days post injection; dpt: Days post transfection; EBV: Epstein-Barr virus; EF1α: Elongation factor 1 promoter; EGFP: Enhanced green fluorescentprotein; EGFP Luc: EGFP-luciferase fusion protein; hCMV: Human cytomegalovirus; HPLG: Haptoglobin; GAPDH: Glyceraldehyde-3-phosphate dehydrogenase; IEP: Immediate early promoter; IRES: Internal ribosomalentry site; kb: Kilobasepair; LPEI: Linear polyethylenimine; Luc: Luciferase; MFI: Mean fluorescence intensity; MCS: Multiple cloning site; ORC: Origin recognition complex; Ori: Origin of replication; PBS: Phosphatebuffered saline; qPCR: Quantitative polymerase chain reaction; shRNA: Small hairpin RNA; stddev: Standard deviation; SM: Smooth muscle differentiation marker; S/MAR: Scaffold/matrix attachment region; SV40-O/P: Simian virus 40 Ori/promoter

## Competing interests

No competing financial or non-financial interests exist.

## Authors’ contributions

RH cloned all constructs mentioned in this manuscript and performed the in vitro-experiments regarding the tissue-specific replication of the pEpito-contructs. TM performed the in vitro-experiments regarding the tissue-specific expression. FK accomplished the qPCR. All *in-vivo* experiments were realized by TM, BS and MO. EW, HL and AB were involved in discussions. RH and MO drafted the manuscript. All authors read and approved the final manuscript.
